# Enhance nursing students’ reflection through Padlet: an action research

**DOI:** 10.12688/mep.19771.1

**Published:** 2023-10-04

**Authors:** Tharin Phenwan

**Affiliations:** 1 School of Health Sciences, University of Dundee, Dundee, Scotland, UK

**Keywords:** Reflection, Undergraduate, Nurses, Qualitative research, Action research, Padlet, Technology

## Abstract

**Background:** Reflective practice (RP) is encouraged amongst healthcare students, including nursing students. However, students do not have a ‘safe space’ to practice reflection before being assessed. Padlet is an interactive platform that can potentially facilitate students’ reflection via its features that enables anonymous participation, asynchronous participation and collaborative learning environment. This study aims to explore the influence of current reflection teaching method on students’ RP and how Padlet can facilitate students’ RP.

**Methods:** An action research was undertaken with 22 first year nursing students from Feb to May 2023. Participants answered questions anonymously pre-class and post-class in two Padlet boards. The researcher gave constructive feedback and signposted good examples of reflection to participants thus enabling ‘champion’ students to emerge during the process. Anonymous texts from two Padlet boards were analysed using reflexive thematic analysis technique.

**Results:** Three themes were generated: i) Pre-class variable baseline understanding of reflection; ii) Co-constructed reflection and iii) Tools and triggers for reflection. Students joined the study with different presumptions and understanding of reflection, ranging from descriptive understanding of the concept, a total misunderstanding of the concept and in-depth understanding of reflection. They all indicated a changed understanding of reflection post-class and emphasized the benefits of a socially constructed learning process. Participants suggested the use of reflective tools (via reflective models) and triggers (via probing questions and feedback) as useful to facilitate their reflection.

**Conclusions:** This study indicates that the current teaching materials enable students to enhance their understanding of reflection. Nevertheless, students could potentially benefit from tools and triggers that will initiate and support their reflection. To that end, Padlet proves a promising tool to enhance students’ reflection via its function to enable anonymity, asynchronous participation and socially constructed learning environment.

## Introduction

Reflective practice (RP) is a practice of making sense of a situation via reflection and is usually occurs during an unexpected or surprising experience ^1^. RP is encouraged amongst healthcare students since it can enhance students’ metacognition which will lead to better learning outcomes, satisfaction with their learning and better long-term professional performances ^2, 3^. Moreover, RP increases students’ self-awareness and long-term professional self-development ^4^. In the UK, the Nursing and Midwifery Council also suggests the continuous use of RP as one of the strategies to sustain and improve nurses’ professional competence ^5^. Consequently, it is beneficial to enhance and foster the RP process for nursing students.

It is challenging to teach RP to students and appropriately determine their capability to reflect: First, RP is a complex activity which can be taught and demonstrated in various forms such as verbally or in writing. Reflection can also happen in various contexts such as at bedside between students and patient, in a lecture hall or small group discussion ^1, 2, 6^. Yet the assessment of students’ RP tends to focus on their capability to demonstrate their RP in writing. This is problematic because it is challenging to differentiate the students who need more support on their externalisation i.e. their ‘writing skills’ and students who need more support on internalisation i.e., ‘reflection skills’ ^4, 6, 7^. Second, RP is often positioned as an individual activity; the notion of group reflection, social interactions and their impact on individuals reflection skills tend to be undervalued ^1, 2, 6^. Third, writing is often a complex activity. Students need to organise and develop ideas and information while using the accurate vocabulary to deliver their ideas without ambiguity ^8^. As such, without an appropriate examples nor peer-support and scaffolding, students may not be aware of the quality of their writing.

Given that students are incentivised to have good grades, they might emulate certain phrases to emulate reflections to achieve that ^9^. This is challenging to ascertain if students were actually reflecting or became a ‘reflective zombie’ who *‘displays all the outer traits of reflection, without having actually reflected’* (de la Croix and Veen, 2018, p. 394). To investigate these overlapping issues further, it is appropriate to determine how the current teaching method and delivery have influenced students’ RP skill in writing. This will be explored via the use of Padlet. This study aims to explore the influence of current reflection method and materials on students’ RP and how Padlet can facilitate students’ RP.

The research question is: How does Padlet facilitates students capability to demonstrate their reflective writing before and after the reflection teaching session?

## Context

The School where this study was undertaken has several undergraduate nursing programmes: The Ordinary degree, Honours degree and Master degree. Despite various training duration and background, all students will study similar modules in their first year, including the Professional and Academic Skills and Knowledge 1 (PASK 1) module. Currently, RP has been introduced to all students as a part of the PASK1 module and has one designated week for teaching activities related to reflection. Students have access to asynchronous learning materials two weeks prior to the class which consists of reading materials, introduction videos to reflection and probing questions posted in discussion board. During the designated teaching week of reflection, students joined smaller 90-minutes group tutorials (30–35 students per group) and participated in two group activities, focusing on writing a reflective writing.

I noticed from my marking in this module in the past few years that students’ performance regarding their reflective writing was hugely variable. Students who underperformed or failed tended to recite the contents that they deemed appropriate with almost no reflection or produced an incoherent piece of writing. As such, it is unclear if they need further support for their externalisation or internalisation. Conversely, top performers can eloquently reflect in writing yet it is not possible to ascertain if they actually reflect or are one of the ‘reflective zombies’; this led to the conception of the study.

## Methods

This study utilises social constructionist research paradigm. I posit that truths and knowledge are socially constructed and there exist multiple realities ^10, 11^. As such, students will co-construct their learning through their interactions with each other. Padlet was chosen as a platform to support and scaffold this socially-constructed reflective learning process.

Padlet is a platform that allows users to interactively participate in virtual walls and the contents that are posted there ^12^. The platform has been widely used in education with both undergraduate and postgraduate students from various fields due to several advantages ^8, 13– 18^. For this study, the benefits of Padlet include anonymity ^15, 16^ which enhanced students’ learning process without any fear of repercussion or from asking ‘stupid question’ ^18, 19^. Next, Padlet enables asynchronous engagement, meaning that students can choose their preferred time and location to study the materials thus fostering a more inclusive learning environment. Moreover, Padlet promotes a supportive and collaborative learning environment for students ^14, 15, 19^. Students can learn from the responses of their peers thus improving their own understanding of the subject.

Finally, Padlet is the most appropriate platform currently available at the university that will allow students to develop their reflection skills. It is the only tool that provides a non-threatening, anonymous and inclusive learning space. The process of group learning within Padlet will enhance students’ group RP as they gain insights from others’ answers. Students can learn from their ‘mistake’ and identify ‘ideal answers’ from their peers. This process should, subsequently, enhance students’ individual RP ^2, 4^. Table 1 summarises the benefits of Padlet over other learning tools within the university.

**Table 1.** Benefits of Padlet over other learning tools.

Learning tools	Benefits that support students’ reflection			
	Anonymous participation	Facilitate inclusive peer-learning space	Asynchronous participation	Foster ‘deep thinking’ time
**Padlet**	Y	Y	Y	Y
**Discussion** ** board**	N	N	Y	Y
**Mentimeter**	Y	N -Inhibitive to students who are not technologically adept or can reply quickly	N	N -Mostly fast, non-reflective responses
**Collaborate**	Potentially yes	N	N	Potentially yes

Action Research (AR) approach was utilised under this social constructionist research paradigm. AR is a cyclical approach for researchers to evaluate and investigate their works which often lead to tangible actions from the research within the higher education contexts ^20, 21^. The study gained an ethical approval from the Skills Hub Research Ethics Committee in December 2022. Convenience sampling technique ^22^ was used to recruit first year nursing students in the academic year 22–23. An advert about the study was circulated via email by an administrator team member to the first year students to ensure that they were not coerced to join the study and that the participation was voluntary. Participants information sheets and informed consent forms were sent to students who were interested to participate via emails. Those who consented to join the study were provided instructions on how to use Padlet boards along with the link to the first Padlet board before the reflection teaching week. Guided questions were posted on the first Padlet board to facilitate participants’ answers and determine their pre-class understanding of reflection (see *Extended data* for the questions asked ^23^). Participants then wrote their answers anonymously and could edit their answers as often as they preferred. The pre-class board became a read-only board once the teaching week of the introduction to reflective practice commenced.

Participants were encouraged to engage with the asynchronous materials provided within the PASK1 module and attended the face-to-face tutorial sessions before they proceeded with this study. The link to the second Padlet board was sent to participants once the reflection teaching week finished. Guided questions were also posted on the second Padlet board to determine participants’ understanding of reflection post-class. I provided constructive feedback to students’ answers and asked them to expand unclear answers as necessary; they had no obligation to reply. Ideal, constructive reflective answers were ‘liked’ to enable champions to emerge from this process and signposted good examples of reflective practice to other students. No new insights were generated after fourteen participants wrote their answers.

## Ethics

The study gained an ethical approval from the ethical committee in the University. Written informed consent were sought before the data generation process.

## Data analysis process

Anonymised pre-class texts and post-class texts were uploaded into Atlas.Ti. I analysed the texts using Reflexive Thematic Analysis approach ^24^. The analytical process involved six recursive phases:

- Familiarising myself with the dataset
  - Texts in both padlet boards were read and reread along with my memos to interpret what participants expressed (semantic meaning) or wanted to express (latent meaning).
- Coding
  - Initial codes from the texts were coded and compared to Gibb’s six stages of reflective cycle: Description; Feelings; Evaluation; Analysis; Conclusion and Action plan ^2^.
- Generating initial themes
  - Initial themes were developed and analysed deductively, linking to Gibb’s reflective cycle. This model was chosen to determine the students’ RP skill pre-class and post-class since it is one of the two recommended reflection models for nursing students and has been widely used in education due to its practicality to ascertain healthcare students’ capability to reflect ^2^.
- Developing and reviewing themes
  - Themes were reread and examined for additional insights.
- Refining, defining and naming themes
  - Themes were revised to make them more meaningful. All themes were refined to make them more reflective to the findings.
- Writing up

## Researcher reflexivity

I am a family doctor (general practitioner) and have been working as a lecturer for more than 10 years. My research and teaching focus are medical education and experiential learning. I teach the concept of reflection regularly with healthcare students including medical students, nursing students, and beyond in both undergraduate and postgraduate levels thus have a good understanding of the concept. I conducted this study as a part of the professional development that I undertook. Consequently, I positioned myself as an ‘insider’ due to these backgrounds as well as my current position as a lecturer at the university.

## Results

From February to May 2023, 22 students out of 495 joined the study and wrote in the first Padlet board; 16 students continued to contribute in the second Padlet board. Three themes were generated: i) Pre-class variable baseline understanding of reflection; ii) Co-constructed reflection and iii) Tools and triggers for reflection. Verbatim texts were used to expand and facilitate the discussion under each theme.

### Pre-class variable baseline understanding of reflection

Participants joined this project with different presumption and understanding of reflection. Their understanding could be rather descriptive or a misunderstanding of the concept:

*‘Reflection is thinking and writing down how I feel about a situation or event at work, home, placement or anywhere.*’

Conversely, some expressed a more in-depth understanding of the concept:

*‘[reflection is]… the ability to look back on a time, event, situation and consider factors which made it a positive, negative situation and how to improve things in order to move on from it and determine how things could positively be changed for future events.’*

This variable understanding might be partially explained by the diverse background of the first-year students which included mature students, students who already have a university degree or students who joined the nursing programme right after they had finished their schools. As such, they all would have different exposure and experiences around reflection. Nevertheless, all participants expressed similar expectation that they would have a different understanding of reflection post-class.

### Co-constructed reflection

All participants expressed a different understanding of reflection post-class; that is, they understood that reflection could happen in any situations and that they could reflect from non-negative experiences as well as how to ensure similar outcomes. This finding differed from the first theme since most participants indicated that they tended to ‘ignore’ their feelings when they reflect or focus mainly on negative experiences. The majority of participants also suggested how they had learnt through others’ answers thus indicating the co-constructed learning element within Padlet boards:

*‘…I agree with a couple of the other comments. I saw reflection as something which is done after a negative experience but I can see now that it can be used following any kind of experience. Why was something good, why did that situation turn out so well, how can we repeat that outcome.’*

This new co-constructed understanding was also reflected in their answers when they explained how they would apply the concept in the future beyond the learning context, resonating with the Action stage of Gibb’s reflective cycle:

*‘By reflecting frequently and using a journal for reflective practice which will help recognise thought patterns, attitudes, behaviours, emotions etc. This information can help improve how I personally deal with future situations/events professionally.’*

### Tools and triggers for reflection

The findings suggested that participants seemed to become aware that reflection models could be used as a tool to facilitate their reflection process:

*‘It [how I reflect post-class] has changed because i didnt know there are models of reflection . I also learned there is alot of emphasis on feelings in a situation.’*

This finding resonates with the literature since students found structured approach more beneficial to enhance the reflection process and their writing ^2, 12, 25^. As such, the use of reflective model should be further utilised in this module and beyond. Moreover, certain triggers seemed to enable participants to express their reflections. Such triggers were probing questions that I outlined in both Padlet boards and my comments:

*[Q: how would you use reflection to facilitate your learning?]*

*‘…To try and reflect regularly, using Rolfe's model […] Examples might be reflecting on why I'm apprehensive about starting difficult subject matter and procrastinating over essays...’*

The findings suggested that both the reflective tools and triggers from educators could potentially be beneficial to foster students’ reflection. This might relate to the scaffolding process in learning where educators could support the students and their current capabilities ^4, 26^.

### Feedback from participants

The majority of participants (14/16) expressed positive feedback in relation to the use of Padlet to facilitate their learning of reflection. Two advantages were indicated by participants. They preferred the asynchronous element of Padlet and seemed to allow them to be more engaged with the process:

*‘I like that you can take your time to put your ideas down.’*

Moreover, the aspect of socially constructed learning was suggested by several participants when they mentioned how their reflection improved after reading other’s answers, including my feedback:

*‘I have really enjoyed reading other peoples views, and how they reflect. I have found it very interesting how we each experience situations differently and have a different outlook in general. I believe I am gaining knowledge from others reflecting and glad I joined this study. Thank you’*

## Discussion

Findings from the study resonate with the literature and suggest that Padlet is conducive to enhance students’ reflection and also their capability to demonstrate their reflective writing. That is, participants strongly preferred the notion of being anonymous. This is beneficial for the RP process which is often a personal recount of students’ poignant experience. As such, the anonymity in Padlet enables them to participate without any fear of being perceived as ‘stupid’ or have reflection publicly ‘invalidated’. The anonymity also increased students’ engagement with each other thus enabling them to create a more supportive and collaborative learning environment ^14, 15, 19^.

Another benefit of Padlet is the asynchronous aspect which allows participants to contribute the study anytime. Although findings do not indicate this benefit, it can be implied that Padlet is useful to foster - or force - thinking time for students over other available learning tools. For instance, Mentimeter is another popular education tool that is widely used in higher education due to its interactivity and engagement with students ^27^. Yet Mentimeter might not be fully useful for reflection since students would need more ‘thinking time’ to conceptualise and making sense of their reflection which is not likely to occur spontaneously.

Finally, the notion of socially-constructed learning environment was strongly indicated by participants. I could provide constructive feedback to participants without knowing their identity. This is useful for participants to identify ‘ideal’ constructions of reflective writing hence the process should scaffold their learning and also empowered ‘champion’ students for their contribution ^26^. The socially-constructed learning environment was further emphasised when participants and I could ‘like’ certain answers to indicate preferred examples of insightful reflective writing thus further validating their contribution. As a result, the process of knowledge co-construction should enhance students’ deep cognitive engagement which proves beneficial for their future academic mastery ^14^.

Findings also strongly indicated that participants are not ‘reflective zombies’. That is, they have no incentive to get good grade from this study yet they meaningfully shared their reflections in writing based from their learning or clinical experiences. As such, the focus to facilitate students’ reflection should be how we can enhance their ability to reflect. Based on the findings, students seem to have a better reflection with the use of reflection tools (e.g., Gibb’s or Rolfe’s reflective models) and triggers from me (via structured questions in Padlet and my comments and feedback) hence this practice could be continued and encouraged.

## Strengths and limitations of the study

To my knowledge, this is the first study to determine the influence of Padlet over nursing students’ RP. The action research approach via Padlet also enables rich depictions of how students co-construct their understanding of reflection in a non-threatening environment thus offering more insights on how we can empower students to reflect. Nevertheless, the study also poses several limitations:

I had a very low participation rate (22 participants out of 495). This limitation might stem from the restricted timeframe for this project in Semester 2. During that time, the students were doing their clinical placement outside the university hence might be less inclined to participate. However, since the study employed the qualitative approach, the numbers of participants were less relevant; participants also provided relatively insightful writing that contributed to meaningful analysis afterwards. Moreover, the concept of information power, suggested by Malterud *et al*. (2016), was applied to ensure the rigour of the findings ^28^. They indicate that the *‘larger information power the sample holds, the lower N is needed and vice versa’* (Malterud *et al*., 2016, p.2). Given that this study has a very specific focus and research questions, the sample size needed was smaller.

Next, I positioned myself as an insider. This insider positionality might influence how I engaged and analysed the data ^29, 30^. I mitigated this issue by keeping track of written memos. The focus was to examine how I - as an insider - analysed certain aspects from the data. I also iteratively immersed with the datasets, as a part of the reflexive thematic analysis technique, to ensure that the final findings were robust.

Finally, due to the nature of action research that focuses on practical issues of the researchers, the findings might be less generalisable ^20, 31^. However, parts of the findings can still be conceptually transferable due to the underlying theory that has been utilised ^32^.

## Future implications for practice

Findings was disseminated to stakeholders within the School via academic seminars and meetings. Proposed changes for teaching reflection include revision of learning materials to ensure that the materials are customised to students with various background. All students will have mandatory learning materials. Students who enrolled under the Honour and Master degrees will have additional learning materials that are customised to their levels. Padlet is also proposed to be used as a tool to teach and facilitate reflection in the upcoming academic year.

## Future implications for research

Future studies could be designed as a longer longitudinal study and explore the changed understanding of students’ reflection over time. To enable students who come from diverse background, future studies could also examine the different students needs especially amongst mature students whose learning strategies and barriers to successful learning differ from non-mature students ^33^.

## Conclusions

This study indicates that the reflection materials in the PASK 1 module seems to enable students to improve their understanding of reflection. Nevertheless, students could potentially benefit from tools and triggers that will initiate and support their reflection. To that end, Padlet is a platform that can potentially facilitate and enhance nursing students’ reflection skills. The benefits of using Padlet as a learning platform, as indicated by participants, mirrors the literature. That is, they overwhelmingly expressed positive feedback for Padlet, especially how they can learn and improve their understanding of reflection from others via anonymised answers and social interactions.

## Data Availability

Even though de-identified, the raw data transcripts of this qualitative study are not publicly available due to information that could compromise the privacy of research participants and as such requests for the data must be approved by Research Ethics Committee before access can be obtained. Methods described in this paper will allow the reader to emulate the study in their own setting. The data that support the findings of this study are available on request from the corresponding author, [TP]. Discovery: Pre-class and post-class questions.pdf. https://doi.org/10.15132/10000239 ^23^. Data are available under the terms of the Creative Commons Attribution 4.0 International license (CC-BY 4.0).
