# Supplementary material for: Enhancing nursing students’ reflection through Padlet: an action research
Source: MedEdPublish (2016). 2023 Dec 28;13:204. Originally published 2023 Oct 4. [Version 2] doi: 10.12688/mep.19771.2 (PMC10792267; doi:10.12688/mep.19771.2)
Supplement: Peer review history

# Reviewer report: Reviewer response for version 2

**DOI:** 10.21956/mep.21533.r35718

**Published:** 2024-02-22

**Authors:** Alison Ledger

**Affiliations:** 1 Academy for Medical Education, Medical School, The University of Queensland, Brisbane, Queensland, Australia

This paper is much improved with further detail and justification, thank you.  The articulation of the themes is also now more consistent with a reflexive thematic analysis approach.

 In future writing, I encourage you to not only explain **who **you were in this research, but also **how** this position may have influenced the research process in positive or negative ways.  You could be a little more explicit about your assumptions/expectations and whether these were reflected in the research findings.

 I still have reservations about this being presented as an action research study.  Instead, it appears to be a scholarly evaluation of the use of padlet to enhance nursing students' understanding of reflection.

 However, on balance, I think this paper will be useful to others who are grappling with the persistent challenge of how to promote reflective practice.  Padlet may offer a way to enable students to collaboratively develop their understandings of reflection.  I thank you for sharing your practice with others.

Have any limitations of the research been acknowledged?

Partly

Is the work clearly and accurately presented and does it cite the current literature?

Partly

If applicable, is the statistical analysis and its interpretation appropriate?

Not applicable

Are all the source data underlying the results available to ensure full reproducibility?

Partly

Is the study design appropriate and is the work technically sound?

Partly

Are the conclusions drawn adequately supported by the results?

Partly

Are sufficient details of methods and analysis provided to allow replication by others?

Partly

Reviewer Expertise:

I am an experienced medical education academic, with particular expertise in qualitative research methods.  I have also taught and assessed reflective writing across different institutions.

I confirm that I have read this submission and believe that I have an appropriate level of expertise to confirm that it is of an acceptable scientific standard, however I have significant reservations, as outlined above.

# Reviewer report: Reviewer response for version 2

**DOI:** 10.21956/mep.21533.r35717

**Published:** 2024-01-16

**Authors:** Rosanne Coutts

**Affiliations:** 1 Southern Cross University, Lismore, New South Wales, Australia

I have reviewed  Enhancing nursing students’ reflection through Padlet: an action research. The authors have made a number of changes that have enhanced the work, particularly providing further justification for the work. The themes have been renamed and, in my view, present a clearer depiction of the outcomes.  This research project supports the development of reflective practice and demonstrates a methodology or ‘how to’ that encourages student learning in nursing.

 To consider: some typos:

- Typo - To his knowledge, this is the first study to determine the influence of Padlet over nursing students’ reflective practice.
- Second, reflective practice is often positioned as an individual activity; the notion of group reflection, social interactions and their impact on individuals reflection skills tend to be undervalued ^1^

Replace with – 'impact on an individual’s reflective skills, tend'.

  

- The researcher noticed from his marking in this module in the past

Change to – 'researcher noticed when assessing in this module'.

  

- Remove this word - 'hugely'.
- Change and have been working to 'and has been working'.

Have any limitations of the research been acknowledged?

Yes

Is the work clearly and accurately presented and does it cite the current literature?

Partly

If applicable, is the statistical analysis and its interpretation appropriate?

Yes

Are all the source data underlying the results available to ensure full reproducibility?

Partly

Is the study design appropriate and is the work technically sound?

Yes

Are the conclusions drawn adequately supported by the results?

Yes

Are sufficient details of methods and analysis provided to allow replication by others?

Yes

Reviewer Expertise:

Teaching and Learning

I confirm that I have read this submission and believe that I have an appropriate level of expertise to confirm that it is of an acceptable scientific standard.

# Reviewer report: Reviewer response for version 1

**DOI:** 10.21956/mep.21185.r35183

**Published:** 2023-12-04

**Authors:** Kim Walker

**Affiliations:** 1 University of Aberdeen, Aberdeen, Scotland, UK

This is an interesting study as reflective practice (RP) is sometimes a difficult area to teach and research. The introduction highlighted the complexity of assessing RP in terms of reflection and/or writing skills. However there are some areas in the paper which would benefit from greater clarity and depth of information.  

 It would have been helpful to have a fuller explanation of why this was considered action research and perhaps why this route was chosen.

 It is unclear why Padlet was chosen as the key conduit. There was some mention about the use of Padlet in the University but further clarity regarding its general use may help in terms of the the recruitment of participants. This would strengthen the paper and methodology section and perhaps give some insight into the participant numbers. If it was a new tool, then this could impact on those volunteering compared with being a common tool that everyone is familiar with. There is some mention in the discussion around other methods, e.g. Mentimeter but it was only fleeting and further discussion on this would be of benefit. 

 You state you are an "insider" but again more clarity around this would be beneficial as you seem to suggest it is due to your background and role as a lecturer rather than an active participant.  

 It is unclear from the results the extent of the benefit of Padlet compared with the standard teaching. Were the same types of activities carried out in the group activities?

 Thematic analysis can be undertaken in several ways and Braun & Clarkes approach in often used. However, it is unclear why Gibbs six stages of reflective cycle were used for the coding since outputs should reflect the research questions. Qualitative data can be themed in different ways and hence the results presented are only your views. It is beneficial to have any analysis looked at a by a third party as part of a sense check. Further information on the themes in the results would strengthen this paper.

Have any limitations of the research been acknowledged?

Partly

Is the work clearly and accurately presented and does it cite the current literature?

Partly

If applicable, is the statistical analysis and its interpretation appropriate?

Not applicable

Are all the source data underlying the results available to ensure full reproducibility?

Partly

Is the study design appropriate and is the work technically sound?

Partly

Are the conclusions drawn adequately supported by the results?

Partly

Are sufficient details of methods and analysis provided to allow replication by others?

Partly

Reviewer Expertise:

I have significant experience in clinical education research.

I confirm that I have read this submission and believe that I have an appropriate level of expertise to confirm that it is of an acceptable scientific standard, however I have significant reservations, as outlined above.

# Response

**Published:** 2023-12-18

**Authors:** Tharin Phenwan

**Affiliations:** Health Sciences, University of Dundee, Dundee, Scotland, UK

1. It would have been helpful to have a fuller explanation of why this was considered action research and perhaps why this route was chosen.   I have added more contextual information in the context as well as methods sections: ‘…The focus of the study was on writing activities since the majority of student’ assessments will be via their writing. Additionally, this focus relates to the requirement from the Nursing and Midwifery Council which will assess nurses reflective account in writing. Padlet was chosen as a platform to support and scaffold this socially-constructed reflective learning process.’ 2. It is unclear why Padlet was chosen as the key conduit. There was some mention about the use of Padlet in the University but further clarity regarding its general use may help in terms of the the recruitment of participants. This would strengthen the paper and methodology section and perhaps give some insight into the participant numbers. If it was a new tool, then this could impact on those volunteering compared with being a common tool that everyone is familiar with. There is some mention in the discussion around other methods, e.g. Mentimeter but it was only fleeting and further discussion on this would be of benefit.    I have added more contextual information in the context as well as methods sections:     ‘…The focus of the study was on writing activities since the majority of student’ assessments will be via their writing. Additionally, this focus relates to the requirement from the Nursing and Midwifery Council which will assess nurses reflective account in writing. Padlet was chosen as a platform to support and scaffold this socially-constructed reflective learning process.’ ‘…During the study conception stage, the researcher asked the PASK 1 module lead as well as the School’s programme leads regarding the justification and relevancy of this project; all agreed with the justification of this project as well as the use of Padlet. Students were contacted via the year representative regarding their opinions of the project; none replied.’ 3. You state you are an "insider" but again more clarity around this would be beneficial as you seem to suggest it is due to your background and role as a lecturer rather than an active participant.   -I have added more texts to clarify this point: ‘…The use of Padlet to facilitate students’ reflective practice was based on the initial review of the literature and the thorough considerations of the available teaching tools at the University (see Table 1). Moreover, the researcher is a team member of the PASK1 module. He is involved with the design of weekly teaching methods, teaching materials and marking. He also led some of the small group tutorials. Consequently, he positioned himself as an ‘insider’ due to these backgrounds as well as his current position as a lecturer at the university.’   4.It is unclear from the results the extent of the benefit of Padlet compared with the standard teaching. Were the same types of activities carried out in the group activities? The study was not designed to be a comparative study of the traditional teaching activities vs Padlet activities. Rather, it is to determine how does the current teaching materials and activities influence students’ RP. And Padlet was chosen as a tool to capture that.   -I also added more information regarding group activities for clarifications: ‘… Students were split into smaller groups. The first activity focused on writing and their reflective accounts. Written examples were provided along with explanation of which examples were considered reflective writing. The second activity was based on students’ recent clinical placement experiences. Guidance and probing questions were provided to enable them to reflect on their experiences. After these two activities, the tutors discussed with students and provided strategies and tools to facilitate reflection as well as its relevancy with wider professional development.’   5. Thematic analysis can be undertaken in several ways and Braun & Clarkes approach in often used. However, it is unclear why Gibbs six stages of reflective cycle were used for the coding since outputs should reflect the research questions. Qualitative data can be themed in different ways and hence the results presented are only your views. It is beneficial to have any analysis looked at a by a third party as part of a sense check. Further information on the themes in the results would strengthen this paper. The initial analysis was to deductively approach the data and compare them to Gibb’s reflective cycle. During the later stages, it was clear that the initial themes were unable to meaningfully capture the findings hence themes were subsequently renamed: ‘…During this stage, the initial themes – based on Gibb’s reflective cycle –  were unable to meaningfully capture the findings nor fully answered the research question. Consequently, all themes were refined to make them more reflective to the findings.’

# Reviewer report: Reviewer response for version 1

**DOI:** 10.21956/mep.21185.r35177

**Published:** 2023-12-04

**Authors:** Rosanne Coutts

**Affiliations:** 1 Southern Cross University, Lismore, New South Wales, Australia

Thank you for the opportunity to review and make comment upon the research manuscript entitled: Enhance nursing students’ reflection through Padlet: an action research.

 Reflective practice is an integral skill for clinical learning, both as a student and for all professionals responsible for patient care. The work represents a reflective practice learning activity with a small group of nursing students. Particular focus is given to the use of Padlet as an interactive program where students can without ‘pressure,’ practice their reflections prior to formal assessment. Thematic or qualitative approaches were taken to analysis. The complexities of the enactment of the skill of reflective practice were well considered in the introduction. Important to note that the actual skill was focused on and evaluated as a written activity rather than a verbal or intuitive process. Therefore, reflective skills were being assessed via written activities only. The section on the context clearly presented the issues about actually knowing how well students are engaging in meaningful reflective practice. The analysis was well considered and outlined. The co-constructed reflection section was well considered and presented. Overall interesting research about an important and relevant lifelong skill.

 Further considerations and suggestions.

1. Suggest the 1 ^st^ sentence of the introduction could be reconsidered or even added to. There are better descriptors that communicate the importance and relevance of reflective practice. Also there is a typo within the sentence.

*Reflective practice (RP) is a practice of making sense of a situation via reflection and is usually occurs during an unexpected or surprising experience.*

1. Title – suggest ‘Enhancing’ rather than ‘Enhance’.
2. The words ‘reflective practice’ and ‘reflective’ are being used interchangeably. Suggest they are different. Would review usage.
3. Suggest ‘reflective practice’ could be in the key words.
4. Some sentences have repeat words. Suggest reviewing the work for these. For example:

*Yet the assessment of students’ RP tends to focus on their capability to demonstrate their RP in writing.*

1. Would use the full form of reflective practice throughout in preference to RP.
2. Suggest the writing be reviewed and all sections written in the 1 ^st^ person changed into ‘the researcher’. This would improve the quality of the writing.  
3. Some further nursing specific literature for your consideration. These would enhance the manuscript and provide deeper definitions and understanding:

- Burns and Bulman (2000 ^1^).
- Clouder (2000 ^2^).
- Hong and Chew (2008 ^3^).
- Lane and Roberts (2022 ^4^).
- McNeill *et al.* (2010 ^5^).
- Williams and Lowes (2001 ^6^).

Have any limitations of the research been acknowledged?

Yes

Is the work clearly and accurately presented and does it cite the current literature?

Partly

If applicable, is the statistical analysis and its interpretation appropriate?

Yes

Are all the source data underlying the results available to ensure full reproducibility?

Partly

Is the study design appropriate and is the work technically sound?

Yes

Are the conclusions drawn adequately supported by the results?

Yes

Are sufficient details of methods and analysis provided to allow replication by others?

Yes

Reviewer Expertise:

Teaching and Learning

I confirm that I have read this submission and believe that I have an appropriate level of expertise to confirm that it is of an acceptable scientific standard, however I have significant reservations, as outlined above.

## References

[1] : Student’s perspectives on reflective practice In S. Burns & C. Bulman (Eds.), Reflective practice in Nursing. *Blackwell Science Ltd.* .2000;

[2] : Reflective Practice: Realising its potential. *Physiotherapy* .2000;86(10) : 10.1016/S0031-9406(05)60985-6 517-522 10.1016/S0031-9406(05)60985-6

[3] : Reflective practice from the perspectives of the bachelor of nursing students in International Medical University (IMU). *Singapore Nursing Journal* .35(5) :5-15 Reference source

[4] : Contextualised reflective competence: a new learning model promoting reflective practice for clinical training. *BMC Med Educ* .2022;22(1) : 10.1186/s12909-022-03112-4 71 10.1186/s12909-022-03112-4 35093060 PMC8801113

[5] : First year specialist trainees' engagement with reflective practice in the e-portfolio. *Adv Health Sci Educ Theory Pract* .2010;15(4) : 10.1007/s10459-009-9217-8 547-58 10.1007/s10459-009-9217-8 20058073

[6] : Reflection: possible strategies to improve its use by qualified staff. *Br J Nurs* .10(22) : 10.12968/bjon.2001.10.22.9332 1482-8 10.12968/bjon.2001.10.22.9332 11842464

# Response

**Published:** 2023-12-18

**Authors:** Tharin Phenwan

**Affiliations:** Health Sciences, University of Dundee, Dundee, Scotland, UK

1. Suggest the 1 ^st^ sentence of the introduction could be reconsidered or even added to. There are better descriptors that communicate the importance and relevance of reflective ****practice. Also there is a typo within the sentence. *Reflective practice (RP) is a practice of making sense of a situation via reflection and is usually occurs during an unexpected or surprising experience.* -I have rephrased the first sentence and also have added additional references. 2. Title – suggest ‘Enhancing’ rather than ‘Enhance’. The title has been renamed as suggested 3. The words ‘reflective practice’ and ‘reflective’ are being used interchangeably. Suggest they are different. Would review usage The phrase reflective practice has been added in the key words as suggested 4.  Some sentences have repeat words. Suggest reviewing the work for these. For example: *Yet the assessment of students’ RP tends to focus on their capability to demonstrate their RP in writing.* Several sentences were restructured for better readability as suggested. 5.  Would use the full form of reflective practice throughout in preference to RP. The phrase reflective practice has been added and used throughout 6. Suggest the writing be reviewed and all sections written in the 1 ^st^ person changed into ‘the researcher’. This would improve the quality of the writing.   This has been changed as suggested. 7. Some further nursing specific literature for your consideration. These would enhance the manuscript and provide deeper definitions and understanding Thank you for these suggestions. I have added recent literature in the introduction section.

# Reviewer report: Reviewer response for version 1

**DOI:** 10.21956/mep.21185.r34984

**Published:** 2023-10-30

**Authors:** Alison Ledger

**Affiliations:** 1 Academy for Medical Education, Medical School, The University of Queensland, Brisbane, Queensland, Australia

Thank you for the opportunity to read your manuscript. The introduction captured the complexities of fostering students' reflective practice well and cited key literature in this area. The focus on padlet emphasised the value of students co-constructing understandings of reflection in a safe environment, which is a useful finding for other educators grappling with how best to include reflection in their curricula. 

 I have several suggestions for making this manuscript even better.

1. Methodological framing. The work is presented as an action research study. Action research tends to be participatory and collaborative and it is unclear who the project collaborators were in this case. Perhaps the collaborators were the students but this was not made explicit. I suggest the project may be better framed as a scholarly exploration or evaluation project, or a student-staff partnership project. If you do this, perhaps the question could be presented as 'How *can *Padlet foster reflective writing among nursing students?'
2. Further consideration of ethical issues beyond informed consent. If I have understood correctly, only 22 out of 495 students volunteered for the study and were accessing and interacting with the padlet. Could the padlet and additional feedback be perceived as an advantage for these students in developing reflective practice, over the 473 students who did not have access to this support? How was this risk mitigated?
3. Further detail about your own position in this research. Though you presented yourself as an 'insider', what this meant in practice could be made a little more explicit. Were you the lead for the PASK1 module? Did you facilitate any of the small group tutorials? What led you to Padlet and what did you expect to find through this project? 
4. More detail about the reflective practice education more broadly. What were the intended learning outcomes? What topics were covered in the readings provided to students? What happened in the group tutorials and activities? Why was the focus on reflective writing when the limitations of this were acknowledged in the background section of the manuscript? How was reflective practice assessed? Addressing these questions is important so the reader can consider what outcomes could be attributed to Padlet, and what could be attributed to the influence of the wider programme of education. Addressing these questions may also enable consideration of additional design limitations.
5. Refinement of the theme titles so that they align with the evaluation question presented and are more consistent with Braun and Clarke's reflexive thematic analysis approach. In addressing the question of how does padlet help? I wonder whether some possible theme titles could be: Uncovering variation in students' baseline understandings of reflection, Co-constructing understandings of reflection, and Prompting reflective practice through tools and triggers. These are just suggestions to explain what I mean, feel free to craft theme titles that make sense to you. One further piece of advice would be to think about whether material in the section 'feedback from participants' could be incorporated within the three themes.
6. Ensure that your discussion points are supported by the content in your results section. The first point about the benefits of anonymity are currently not captured in your results section. Were there any quotes from the Padlet that could support this claim? I also wondered whether the first theme could be discussed in greater depth - is there value in knowing students' levels of understanding when they enter training?
7. Consider wider implications beyond Padlet. Though your study was about Padlet, are there other ways that educators could encourage co-construction of understandings and safe ways of developing reflective practice?

I hope you find this feedback helpful and encourage you to continue developing this work.

Have any limitations of the research been acknowledged?

Partly

Is the work clearly and accurately presented and does it cite the current literature?

Partly

If applicable, is the statistical analysis and its interpretation appropriate?

Not applicable

Are all the source data underlying the results available to ensure full reproducibility?

Partly

Is the study design appropriate and is the work technically sound?

Partly

Are the conclusions drawn adequately supported by the results?

Partly

Are sufficient details of methods and analysis provided to allow replication by others?

Partly

Reviewer Expertise:

I am an experienced medical education academic, with particular expertise in qualitative research methods.  I have also taught and assessed reflective writing across different institutions.

I confirm that I have read this submission and believe that I have an appropriate level of expertise to confirm that it is of an acceptable scientific standard, however I have significant reservations, as outlined above.

# Response

**Published:** 2023-12-18

**Authors:** Tharin Phenwan

**Affiliations:** Health Sciences, University of Dundee, Dundee, Scotland, UK

**Comments** **Locations** **Reviewer 1**     Thank you for the opportunity to read your manuscript. The introduction captured the complexities of fostering students' reflective practice well and cited key literature in this area. The focus on padlet emphasised the value of students co-constructing understandings of reflection in a safe environment, which is a useful finding for other educators grappling with how best to include reflection in their curricula.  Thank you for your feedback   Methodological framing. The work is presented as an action research study. Action research tends to be participatory and collaborative and it is unclear who the project collaborators were in this case. Perhaps the collaborators were the students but this was not made explicit. I suggest the project may be better framed as a scholarly exploration or evaluation project, or a student-staff partnership project. If you do this, perhaps the question could be presented as 'How *can *Padlet foster reflective writing among nursing students?' -The main collaborators were the module leader and programme leads. During the study conception, I asked for their opinions in terms of the relevancy of the project, the challenges around reflection teaching. All agreed with the justification of this project as well as the use of Padlet. -I also reached out to few students during the study conception but as discussed in the limitation section, they were at their placements outside the university hence were rather occupied; none replied to my inquiries. I have added more texts in the manuscript to clarify this point: ‘… During the study conception stage, the researcher asked the PASK 1 module lead as well as the School’s programme leads regarding the justification and relevancy of this project; all agreed with the justification of this project as well as the use of Padlet. Students were contacted via the year representative regarding their opinions of the project; none replied.’   -The research question was amended as suggested. Context section Further consideration of ethical issues beyond informed consent. If I have understood correctly, only 22 out of 495 students volunteered for the study and were accessing and interacting with the padlet. Could the padlet and additional feedback be perceived as an advantage for these students in developing reflective practice, over the 473 students who did not have access to this support? How was this risk mitigated? -That is correct. Only 22 consented to join the project. I anticipate that these students may have more short-term benefit since they received additional feedback as well as saw others’ reflective writing. However, all students will not be assessed in reflective writing in this module. They will be assessed in other modules hence the benefits could be minimal e.g., these 22 students may have a better understanding of reflection at the beginning but all should have similar understanding once they have started their reflective practice assignments.  - Further detail about your own position in this research. Though you presented yourself as an 'insider', what this meant in practice could be made a little more explicit. Were you the lead for the PASK1 module? Did you facilitate any of the small group tutorials? What led you to Padlet and what did you expect to find through this project?  -I have added more texts to clarify this points:   ‘… The researcher is a team member of the PASK1 module. He is involved with the design of weekly teaching methods, teaching materials and marking. He also led some of the small group tutorials.’   -‘The use of Padlet to facilitate students’ reflective practice was based on the initial review of the literature and the thorough considerations of the available teaching tools at the University (see Table 1).’ Researcher reflexivity More detail about the reflective practice education more broadly. What were the intended learning outcomes? What topics were covered in the readings provided to students? What happened in the group tutorials and activities? Why was the focus on reflective writing when the limitations of this were acknowledged in the background section of the manuscript? How was reflective practice assessed? Addressing these questions is important so the reader can consider what outcomes could be attributed to Padlet, and what could be attributed to the influence of the wider programme of education. Addressing these questions may also enable consideration of additional design limitations. What were the intended learning outcomes? ‘The reflection teaching week partly addressed two ILOs of the PASK1 module. That is, upon completion of the module, students will be able to: *3. Demonstrate understanding of the need for different academic and professional skills, including professional communication, documentation and person-centred care.* *5. Discuss professionalism in the context of being a nursing student* By the end of the week, students should be able to:

- define reflection
- understand the role of reflection in professional learning
- introduce the idea of reflective practice
- be able to distinguish between reflection and reflective practice
- discuss the role of reflective practice in improving professional effectiveness’

  What topics were covered in the readings provided to students? ‘…Asynchronous study materials included three YouTube videos which focus on reflective practice, reflective writing and critical reflection reading materials on reflective practice, guide to reflective writing and a peer-reviewed rapid review discussing reflective practice in crisis situations.’ What happened in the group tutorials and activities? ‘…Students were split into smaller groups. The first activity focused on writing and their reflective accounts. Written examples were provided along with explanation of which examples were considered reflective writing. The second activity was based on students’ recent clinical placement experiences. Guidance and probing questions were provided to enable them to reflect on their experiences. After these two activities, the tutors discussed with students and provided strategies and tools to facilitate reflection as well as its relevancy with wider professional development.’   Why was the focus on reflective writing when the limitations of this were acknowledged in the background section of the manuscript? ‘The focus of the study was on writing activities since the majority of student’ assessments will be via their writing. Additionally, this focus relates to the requirement from the Nursing and Midwifery Council which will assess nurses reflective account in writing.’ How was reflective practice assessed? Students’ reflective practice will not be marked in the PASK1 module; they will be assessed in other modules in each academic year. Moreover, they will be assessed during their clinical placements by their assessors which will be beyond the scope of this study and the PASK1 module. Context section Refinement of the theme titles so that they align with the evaluation question presented and are more consistent with Braun and Clarke's reflexive thematic analysis approach. In addressing the question of how does padlet help? I wonder whether some possible theme titles could be: Uncovering variation in students' baseline understandings of reflection, Co-constructing understandings of reflection, and Prompting reflective practice through tools and triggers. These are just suggestions to explain what I mean, feel free to craft theme titles that make sense to you. One further piece of advice would be to think about whether material in the section 'feedback from participants' could be incorporated within the three themes. I have added more texts to clarify these points:   ‘…During this stage, the initial themes – based on Gibb’s reflective cycle –  were unable to meaningfully capture the findings nor fully answered the research question’   -I also rename the themes to : i) Unpacking variation in students baseline understanding of reflection; ii) Co-constructed understanding of reflection and iii) Prompting reflective practice through tools and triggers   -The feedback section was incorporated into the existing themes Data analysis process, Results Ensure that your discussion points are supported by the content in your results section. The first point about the benefits of anonymity are currently not captured in your results section. Were there any quotes from the Padlet that could support this claim? I also wondered whether the first theme could be discussed in greater depth - is there value in knowing students' levels of understanding when they enter training? -I have added more quotes to support the benefit of Padlet around anonymity ‘…Padlets provide a  good platform of confidentiality, anonymity and willingness to share.  For learning and understanding reflection, it compliments the class and learning materials, and reading answers and feedback to responses has helped contribute to my understanding.’   -More texts were added under theme 1: ‘…Unfortunately, the teaching materials provided were prepared with an implicit assumption that that students would have a similar understanding of reflection. This is problematic since the existing teaching materials may not be fully useful to students who already have some understanding of the concept.’ Results section Consider wider implications beyond Padlet. Though your study was about Padlet, are there other ways that educators could encourage co-construction of understandings and safe ways of developing reflective practice? -I have added more texts to clarify this points ‘Furthermore, based on the findings, students seem to appreciate the use of Padlet since it enables them to develop their reflective practice anonymously. This is compounded with the co-construction of understanding of their reflection with others. As such, These two perceived benefits – anonymity and co-constructed learning element- should be further utilised beyond the use of Padlet.’ Future implications for research

# Response

**Published:** 2023-12-18

**Authors:** Tharin Phenwan

**Affiliations:** Health Sciences, University of Dundee, Dundee, Scotland, UK

Apologies- I have copied and pasted from a table in Microsoft Words file and the responses are very hard to read. Here they are:   1. Methodological framing. The work is presented as an action research study. Action research tends to be participatory and collaborative and it is unclear who the project collaborators were in this case. Perhaps the collaborators were the students but this was not made explicit. I suggest the project may be better framed as a scholarly exploration or evaluation project, or a student-staff partnership project. If you do this, perhaps the question could be presented as 'How *can *Padlet foster reflective writing among nursing students?' -The main collaborators were the module leader and programme leads. During the study conception, I asked for their opinions in terms of the relevancy of the project, the challenges around reflection teaching. All agreed with the justification of this project as well as the use of Padlet. -I also reached out to few students during the study conception but as discussed in the limitation section, they were at their placements outside the university hence were rather occupied; none replied to my inquiries. I have added more texts in the manuscript to clarify this point: -The research question was amended as suggested. 2. Further consideration of ethical issues beyond informed consent. If I have understood correctly, only 22 out of 495 students volunteered for the study and were accessing and interacting with the padlet. Could the padlet and additional feedback be perceived as an advantage for these students in developing reflective practice, over the 473 students who did not have access to this support? How was this risk mitigated? That is correct. Only 22 consented to join the project. I anticipate that these students may have more short-term benefit since they received additional feedback as well as saw others’ reflective writing. However, all students will not be assessed in reflective writing in this module. They will be assessed in other modules hence the benefits could be minimal e.g., these 22 students may have a better understanding of reflection at the beginning but all should have similar understanding once they have started their reflective practice assignments.  3. Further detail about your own position in this research. Though you presented yourself as an 'insider', what this meant in practice could be made a little more explicit. Were you the lead for the PASK1 module? Did you facilitate any of the small group tutorials? What led you to Padlet and what did you expect to find through this project?  I have added more texts to clarify this points in the researcher reflexivity section: ‘… The researcher is a team member of the PASK1 module. He is involved with the design of weekly teaching methods, teaching materials and marking. He also led some of the small group tutorials.’ -‘The use of Padlet to facilitate students’ reflective practice was based on the initial review of the literature and the thorough considerations of the available teaching tools at the University (see Table 1).’ 4. More detail about the reflective practice education more broadly. What were the intended learning outcomes? What topics were covered in the readings provided to students? What happened in the group tutorials and activities? Why was the focus on reflective writing when the limitations of this were acknowledged in the background section of the manuscript? How was reflective practice assessed? Addressing these questions is important so the reader can consider what outcomes could be attributed to Padlet, and what could be attributed to the influence of the wider programme of education. Addressing these questions may also enable consideration of additional design limitations.   What were the intended learning outcomes? ‘The reflection teaching week partly addressed two ILOs of the PASK1 module. That is, upon completion of the module, students will be able to: *3. Demonstrate understanding of the need for different academic and professional skills, including professional communication, documentation and person-centred care.* *5. Discuss professionalism in the context of being a nursing student* By the end of the week, students should be able to:

- define reflection
- understand the role of reflection in professional learning
- introduce the idea of reflective practice
- be able to distinguish between reflection and reflective practice
- discuss the role of reflective practice in improving professional effectiveness’

  What topics were covered in the readings provided to students? ‘…Asynchronous study materials included three YouTube videos which focus on reflective practice, reflective writing and critical reflection reading materials on reflective practice, guide to reflective writing and a peer-reviewed rapid review discussing reflective practice in crisis situations.’ What happened in the group tutorials and activities? ‘…Students were split into smaller groups. The first activity focused on writing and their reflective accounts. Written examples were provided along with explanation of which examples were considered reflective writing. The second activity was based on students’ recent clinical placement experiences. Guidance and probing questions were provided to enable them to reflect on their experiences. After these two activities, the tutors discussed with students and provided strategies and tools to facilitate reflection as well as its relevancy with wider professional development.’   Why was the focus on reflective writing when the limitations of this were acknowledged in the background section of the manuscript? ‘The focus of the study was on writing activities since the majority of student’ assessments will be via their writing. Additionally, this focus relates to the requirement from the Nursing and Midwifery Council which will assess nurses reflective account in writing.’ How was reflective practice assessed? Students’ reflective practice will not be marked in the PASK1 module; they will be assessed in other modules in each academic year. Moreover, they will be assessed during their clinical placements by their assessors which will be beyond the scope of this study and the PASK1 module.   5.  Refinement of the theme titles so that they align with the evaluation question presented and are more consistent with Braun and Clarke's reflexive thematic analysis approach. In addressing the question of how does padlet help? I wonder whether some possible theme titles could be: Uncovering variation in students' baseline understandings of reflection, Co-constructing understandings of reflection, and Prompting reflective practice through tools and triggers. These are just suggestions to explain what I mean, feel free to craft theme titles that make sense to you. One further piece of advice would be to think about whether material in the section 'feedback from participants' could be incorporated within the three themes.   I have added more texts to clarify these points: ‘…During this stage, the initial themes – based on Gibb’s reflective cycle –  were unable to meaningfully capture the findings nor fully answered the research question’ -I also rename the themes to : i) Unpacking variation in students baseline understanding of reflection; ii) Co-constructed understanding of reflection and iii) Prompting reflective practice through tools and triggers -The feedback section was incorporated into the existing themes 6. Ensure that your discussion points are supported by the content in your results section. The first point about the benefits of anonymity are currently not captured in your results section. Were there any quotes from the Padlet that could support this claim? I also wondered whether the first theme could be discussed in greater depth - is there value in knowing students' levels of understanding when they enter training? -I have added more quotes to support the benefit of Padlet around anonymity ‘…Padlets provide a  good platform of confidentiality, anonymity and willingness to share.  For learning and understanding reflection, it compliments the class and learning materials, and reading answers and feedback to responses has helped contribute to my understanding.’ -More texts were added under theme 1: ‘…Unfortunately, the teaching materials provided were prepared with an implicit assumption that that students would have a similar understanding of reflection. This is problematic since the existing teaching materials may not be fully useful to students who already have some understanding of the concept.’   7. Consider wider implications beyond Padlet. Though your study was about Padlet, are there other ways that educators could encourage co-construction of understandings and safe ways of developing reflective practice? -I have added more texts to clarify this points ‘Furthermore, based on the findings, students seem to appreciate the use of Padlet since it enables them to develop their reflective practice anonymously. This is compounded with the co-construction of understanding of their reflection with others. As such, These two perceived benefits – anonymity and co-constructed learning element- should be further utilised beyond the use of Padlet.’
